# Saratoga in Winter

**Published:** 1875-10

**Authors:** 


### SARATOGA IN WINTER.

If we were not quite well, and felt inclined to visit Saratoga, we are decidedly of the opinion that we would select the fall and winter for the purpose. The crowded, fashionable season, is not the best time for the health-seeker, we are certain. There is too much activity and too little repose; too much excitement and too little quietude; too many interruptions and temptations to late hours and dissipation. We should seek advice and the comforts of a home, at the Remedial Institute of the Drs. Strong. We went all over this establishment last summer, and were charmed with what we saw and heard. We thought we had never seen a more desirable spot to be sick in, though we do not believe a patient would find it an easy place in which to continue sick, because, with the excellent systems employed by the Drs. Strong, and the vast array of remedial appliances, we are at a loss to see how sickness could be greatly prolonged. We talked with several of the patients, and all testified to the great benefits derived from the treatment employed. This, we are glad to say, is of a hygienic character, and involves rational living, the use of water in all forms, and the employment of electricity, the movement-cure, and many other non-medicinal appliances. The Drs. Strong—father and son—are educated physicians, and do not discard medicines in their practice; but they have had so many hundreds of cases in which drugs have been excessively employed, that they are fairly driven to make large use of other remedial agencies. To meet all cases, they have erected a great establishment, which is at once a place for the sick and the well; a charming home, where the air is genial and delightful, the society attractive and intelligent, the table and appointments first-class, and the means for casting out the evil spirit of disease, excellent beyond compare. The waters of the Springs continue to flow at all seasons, and can be enjoyed, fresh from the fountain, without trouble or cost. On the whole, the Remedial Institute at Saratoga strikes us as a most desirable spot, outside of the crowded, fashionable term, and hence we recommend our sick friends, to whom an entire change of scene would prove so salutary, to send to the gentlemen alluded to for circulars explanatory of their modes of treatment.

---

Dr. Van Deren's Prescription.—Messrs. Hall & Ruckel, wholesale druggists, 218 Greenwich Street, inform us that the little sugar granules called "Van Deren's Remedy," for bowel complaint of children, has fairly won its place to public favor during the past summer. The sale was large, and in no single instance was the Remedy referred to, except in terms of the highest praise. Many persons have called, or written for supplies, stating they would not be without it.

Children are liable to attacks of diarrhœa at all seasons, and no other remedy so promptly checks the diarrhœa and removes the cause of it, as this, and no other remedy is so safe and so easily administered, having only the taste of sugar.

Hall & Ruckel have recently put in the market a remedy for piles, which promises to become very popular. It is called "Pile Suppository," and is a favorite prescription of the great French surgeon, Nelaton. If applied on going to bed at night, even in the severest cases, the patient in the morning finds the pain and soreness entirely gone, or greatly relieved. It is the cleanest and most easily applied of all medicated applications for piles which we have seen. The price of each of the above remedies is fifty cents a package. Sent by mail free, on the receipt of price.

